# Detection of *Brucella* spp. in milk from seronegative cows by real-time polymerase chain reaction in the region of Batna, Algeria

**DOI:** 10.14202/vetworld.2018.363-367

**Published:** 2018-03-26

**Authors:** Rabehi Sabrina, Hamdi Taha Mossadak, Mamache Bakir, Meghezzi Asma, Boushaba Khaoula

**Affiliations:** 1 Department of Veterinary Science, Institute of Veterinary and Agronomic Sciences, University of Batna 1, Batna, Algeria; 2 Research Laboratory HASAQ, High National Veterinary School, Algiers, Algeria; 3 Laboratory of Molecular Biology and Microbiology of Constantine Biotechnology Research Center, Constantine, Algeria

**Keywords:** *Brucella* spp, milk, real-time polymerase chain reaction, seronegative cows

## Abstract

**Aim:**

The aim of this study was to detect *Brucella* spp. DNA in milk samples collected from seronegative cows using the real-time polymerase chain reaction (PCR) assay for diagnosis of brucellosis in seronegative dairy cows to prevent transmission of disease to humans and to reduce economic losses in animal production.

**Materials and Methods:**

In this study, 65 milk samples were investigated for the detection of *Brucella* spp. The detection of the *IS711* gene in all samples was done by real-time PCR assay by comparative cycle threshold method.

**Results:**

The results show that of the 65 DNA samples tested, 2 (3.08%) were positive for *Brucella* infection. The mean cyclic threshold values of *IS711* real-time PCR test were 37.97 and 40.48, indicating a positive reaction.

**Conclusion:**

The results of the present study indicated that the real-time PCR appears to offer several advantages over serological tests. For this reason, the real-time PCR should be validated on representative numbers of *Brucella*-infected and free samples before being implemented in routine diagnosis in human and animal brucellosis for controlling this disease.

## Introduction

Brucellosis is one of the most common and economically important zoonoses globally [1]. Bacteria of the genus *Brucella* spp. are coccobacilli, Gram-negative, aerobic, non-spore forming, non-motile, and non-capsulated [2]. To date, twelve different *Brucella* species have been described [3,4]. Each one may infect different host species, but each *Brucella* species has a preference for its host species: *Brucella melitensis* (sheep and goats), *Brucella abortus* (cattle), *Brucella suis* (pigs), *Brucella ovis* (rams), *Brucella canis* (dogs), *Brucella microti* (rodents - *Microtus arvalis*), *Brucella neotomae* (rodents - *Neotoma lepida*), *Brucella pinnipedialis* (pinnipeds), *Brucella ceti* (cetacea), and *Brucella inopinata* (originally isolated from a human patient, but its preferential host is not known) [5-7]. The two most recently described species are *B. papionis*, which was isolated from two baboons with retained placenta [4], and *Brucella vulpis* which was isolated in Austria from the mandibular lymph nodes of two red foxes [3].

Zoonotic transmission occurs most frequently through unpasteurized milk products in urban settings, while occupational exposure of farmers, veterinarians, or laboratory workers can result from direct contact with infected animals or tissues or fluids associated with abortion [8].

Brucellosis in humans almost always originates from an animal reservoir [9]. To reduce the incidence of many zoonotic infections among humans, the pathogens must be controlled in the animal population [10].

Diagnosis is usually based on serological tests and/or cultivation. Serological assays are rapid, sensitive, and easy to perform but lack specificity due to cross-reactions with other bacteria, particularly with *Yersinia enterocolitica* O:9, that result from O chains antigenic similarity [11,12]. Conventional cultural isolation and identification of the agent is the gold standard test for *Brucella abortu*s but time consuming, laborious and also need skills as well as biosafety level-3 laboratory and measures to prevent zoonosis [13].

Due to isolation problems, the significance of molecular-based detection techniques is increasing. Polymerase chain reaction (PCR) techniques for the diagnosis of *Brucella* spp. are used successfully both from different clinical samples and pure cultures [14].

There are also real-time or quantitative PCR (qPCR) assays that have been developed for rapid and safe detection of *Brucella*, including assays targeting the *bcsp31* gene or the *IS711* insertion sequence [15].

In this study, we discuss the extraction and purification of column DNA from raw milk using a specific kit, and we tried to use a reliable molecular procedure that could increase the sensitivity and specificity of the detection of *Bruclla* spp. DNA in bovine milk which detects insertion of the *IS711* sequence of *B. melitensis*, *B. abortus*, *B. canis*, *B. ovis*, and *B. suis*. *IS711* is characteristic of *Brucella* spp. and appears in variable number (5-38 copies) [16].

## Materials and Methods

### Ethical approval

In this investigation, we did not use live animals. Milk samples were collected during routine milking by traditional hand-stripping. Therefore, no ethical approval was needed in the present study.

### Samples of milk

A total of 65 milk samples were taken from seronegative cows (ages 3-8 years) belonging to 3 dairy farms in the Wilaya of Batna: 2 in the district of Djerma and 1 in the district of Ain Yagout. The first farm contains 21 cows, the second 25 cows, and the third 19 cows.

The milk samples were collected aseptically from teats of the udder which had previously been cleaned with water and soap, and then, the surface was sterilized with 70% ethanol. For each animal, the first jet of milk was removed, and then 15 mL of milk were collected from 4 quarters in sterile Falcon tubes previously identified. The samples were immediately transported to the laboratory at +4°C for DNA extraction.

This study was conducted at the Laboratory of Microbiology and Molecular Biology of Constantine Biotechnology Research Center, Algeria, during July 2017.

### Pre-treatment of milk

Initially, the milk undergone a pre-treatment as follows: A test sample of 800 µL of the milk was centrifuged for 5 min at 10,000 g, then the supernatant was removed, and the pellet was resuspended in 300 µL of phosphate-buffered saline.

### Extraction of DNA

Genomic DNA was extracted from raw milk using a BioExtract^®^ Column purification kit according to the manufacturer protocol (Cat N° BEC050).

The extracted DNA is then quantified using Nanodrop^®^ ND 8000.

The DNA was then stored at 4°C until use.

### Real-time PCR amplification

Real-time PCR was used to detect the presence of *Brucella* spp. DNA in milk samples as described in the kit BactoReal^®^ kit *Brucella* spp. (REF: DVEB02113). This test has been developed and validated for ABI PRISM^®^ 7500 (Fast) instrument (Applied Biosystems). This test allows rapid and sensitive detection of DNA *Brucella* spp. from purified milk samples.

BactoReal^®^ Kit *Brucella* spp. detects insertion of the *IS711* sequence of *B. melitensis*, *B. abortus*, *B. canis*, *B. ovis*, and *B. suis. IS711* is characteristic of *Brucella* spp. and appears in variable number (5-38 copies).

A positive internal control of the system for the detection at Cy5 (667 nm) makes it possible to demonstrate the inhibitions of the PCR, resulting in false-negative results when interpreting the results due to the inhibition of PCR in real time.

### The reaction mixture

The real-time PCR assay was performed by an ABI PRISM^®^ 7500 instrument thermal cycler (Applied Biosystems) using 96-well optical barcode plates (Ref: 4306737) and Adhesive Optical Films Starter Kit (Ref: 4311971) following the manufacturer’s instructions (Ingenetix, Austria). The real-time PCR amplification was carried out in a reaction mixture of 20 µL composed of 5 µL of sample (or genomic DNA) containing matrix DNA and 15 µL of Master Mix consisting of 3.0 µL of water, 10 µL of DNA mix reaction (1 µL of *Brucella* Mix Assay) for the detection of *Brucella* spp., and 1 µL of CR mix assay (Primers and Probes (Cy5) for IPC detection).

At least one negative control (water), one positive control (*B. abortus*), and one negative extraction included by PCR were ensured.

According to Ingenetix recommendations, all PCR analyzes are done in duplicate to optimize the probability of detecting pathogens and facilitates the interpretations of results.

### Programming of the temperature profile

The reaction was carried out in a DNA thermocycler (Applied Biosystems) at a preliminary denaturing temperature of the DNA in dry at 95°C, followed by 45 cycles consisting of 5 s at 95°C for denaturation of DNA, and 1 min at 60°C for polymerase-mediated primer extension.

## Results

The results show that of the 65 DNA samples tested, 2 (3.08%) were positive and 63 (96.92%) were negative for brucellosis, based on real-time PCR testing used. The mean cyclic threshold (Ct) values of *IS711* real-time PCR test were 37.97 and 40.48, indicating a positive reaction.

**Figure-1:**
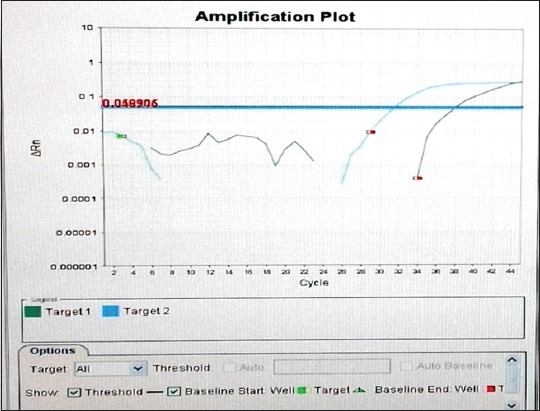
Logarithmic curve of amplification of one of the two positive samples. Target 1: FAM fluorescence signal of *Brucella* spp. DNA. Target 2: Fluorescence signal of the internal positive control (Cy5).

The curve (Figure-1) shows a Target 1 (FAM Signal) multiplication which means that the *Brucella* spp. DNA was amplified and the sample interpreted as a positive. However, the Ct is 37.97 (low value) which means the presence of *Brucella* spp. in small quantities.

**Figure-2:**
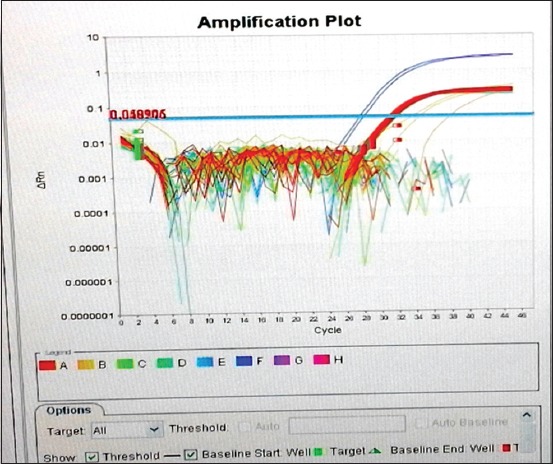
Logarithmic amplification curve of all samples (all plates even positive and negative controls).

The curve (Figure-2) shows a Target 1 multiplication for positive controls (undiluted and diluted), as well as for 2 samples only. For the other samples, the Target 1 multiplication is not detectable (below the threshold value). The curves above the threshold value are Target 2 (Cy5) CPI multiplications.

## Discussion

Brucellosis is an ancient and one of the world’s most widespread zoonotic diseases affecting both public health and animal production [17]. In livestock, the disease results in significant economic losses due to reproductive impairment caused by abortion, stillbirth or weak calves and neonatal mortality, and infertility [5]. In humans, *Brucella* spp. infection causes a febrile disease that may be associated with a broad spectrum of symptoms, and it may be fatal in some cases [18].

In the present study, we investigated the presence of *Brucella* DNA in the milk samples, and we chose to analyze all samples with primers targeting the *IS711* insertion sequence because *IS711* is a specific and highly sensitive method for the safe detection of the genus *Brucella* [15].

This study found that, of the 65 milk samples of seronegative cows, 3.08% (n=2) were qPCR positive for *Brucella* DNA with Ct-values ranging between 37.97 and 40.48. The results obtained in this study confirm that bacterial excretion of milk was low but sufficient to induce infection. The consumption of non-pasteurized dairy products from *Brucella*-infected animals is the most frequent route of human infection in general. Hence, pasteurization of milk will reduce *Brucella* transmission to humans [19].

The prevalence of brucellosis in Algeria in this study was lesser as compared to an earlier study obtained by Islam *et al*. [20], Rajala *et al*. [21], and Hinić *et al*. [22]. They found a similar level of positive cases among their examined herds with 11.23%, 11.8%, and 11.1%, respectively. The discrepancy between the serology and PCR results observed in the current study might indicate that the true number of *Brucella*-infected cattle within the study area could be underestimated by serology screening. False serological negative results have been reported previously [11,23,24] and one explanation could be that antibody titers reduce over time [25]. Hence, seronegative animals in the current study, which tested positive by qPCR, could have been exposed to *Brucella* and turned seronegative after a certain time period. Alternatively, if sampling at an early stage of the infection, i.e., within the first 14 days, the humoral immune response has not yet induced detectable levels of antibodies in the host [26]. Furthermore, individuals infected *in utero* or in the early post-natal period can become latently infected and hence never become seropositive [27]. Approximately 3.5% of infected cows are estimated to deliver latent-infected offspring [28]. In addition, MacMillan [29] reported that the Rose Bengal Test antigen could deteriorate when repeatedly cycled between refrigerator and room temperature during use. However, that serological testing has limits, especially after the disease has entered the chronic phase, when the organism is harbored intracellularly, often in the supramammary lymph nodes and the udder. Indeed, because the most important aspect of *Brucella* ecology is their ability to establish an intracellular replicative niche and remain protected from the host immune responses [2].

If this is the case, it might partially explain the discrepancy between the serology and qPCR results observed in the current study. Hypothetically, the discrepancy between the serology and qPCR results could be caused by previous vaccination against brucellosis as reported from a study in Egypt where cattle vaccinated with *RB51* tested negative by serology tests but positive by qPCR [30]. However, in the current study, the samples of milk were taken from dairy cows submitted a control program of 1995 that none of the cattle had been vaccinated against brucellosis in Algeria.

## Conclusion

Real-time PCR appears to offer several advantages over serological test in detecting the presence of extensive infection in cow’s milk samples. The detection was about 0.25 pg DNA (equivalent to 25-50 cells). As amplicon production is monitored and measured in real-time, results are available within 30 min [24]. This insertion sequence *IS711* appears in several copies (10-40) all over the genome, but distribution and number differ in *Brucella* species [31]. For this reason, it is highly recommended to use this real-time PCR procedure to identify *Brucella* in all types of milk samples. Furthermore, we advise to use this procedure as a regular screening test in farms animals.

## Authors’ Contributions

RS prepared the study design, carried out the research, and analyzed the data under the supervision of HTM and MB. RS and MA executed the extraction of DNA. RS and BK executed the real-time PCR. The manuscript was drafted and revised by RS under the guidance of HTM and MB. All authors read and approved the final manuscript.
